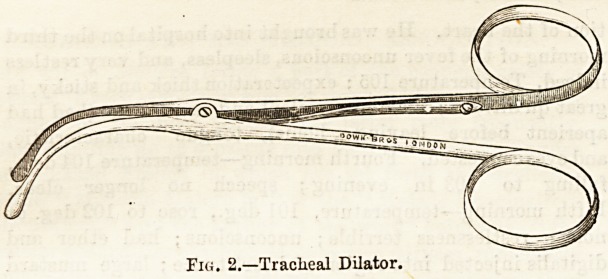# "The Hospital" Nursing Mirror

**Published:** 1897-10-30

**Authors:** 


### "The Hospital" Nursing Mirror.

Being the Nursing Section of "The Hospital."

[Contributions for this Section of "The Hospital" should be addressed to the Editor, The Hospital, 28 & 29, Southampton Street, Strand, London, W.C., and should have the word "Nursing" plainly written in left-hand top corner of the envelope.]

### News from the Nursing World.

### DEATH OF THE DUCHESS OF TECK.

The death of H.R.H. Princess Mary, Duchess of Teck, comes as a personal blow to many, and to none more so than those occupied in ministering to the sick, whose efforts she never failed to encourage. She was universally beloved, and has taken so warm a sympathy in all good work. She was attacked last Monday with her old complaint, and on Tuesday night Dr. Allingham again performed an operation for strangulated hernia. The Princess, however, sank, and died at three the following morning. The Duchess of York had arrived on a visit to her mother some days before.

### MATERNITY AMBULANCES.

Some little time ago the French Press drew attention to the fact that, whereas in Chicago and Moscow ambulances were attached to maternity hospitals, yet those of France were without them. The cause of the outcry was that a fine baby boy entered this mundane sphere in an omnibus. It is undoubtedly convenient to blame the hospitals; they fill the same place to the general public in matters medical as the proverbial "cat, dog, or baby" do in domestic catastrophes; but it seemed probable that here the blame lay further afield. Inquiries at Queen Charlotte's Hospital proved this to be the case. Last year out of some 1,200 births none took place outside the walls, and during the year before only one occurred. In this case the mother was immediately put into a cab and driven to the hospital, which she reached in time to be attended by the sister in charge. Ambulances cannot be of the slightest service. Prudent forethought on the part of the patient is the remedy needed.

### CRUMPSALL NURSES' HOME.

Crumpsall Workhouse Infirmary is one of the largest in the kingdom. It was built about twenty years ago, and had then accommodation for nearly 1,400 patients and an adequate nursing staff. As time went by the Manchester Board of Guardians abolished the pauper nurse before the Local Government Board meddled in the matter, and provided for more nurses by fitting up a ward with cubicles. A new home has been erected, with sleeping accommodation for forty nurses, at a cost of £4,000. The Lord Mayor of Manchester, J. T. Roberts, Esq., opened it on October 13th. The nursing staff numbers nearly 100, who, with very few exceptions, have been trained at the infirmary. The ward used by the nurses now reverts to its original purpose, which, in view of the labour disputes entailing so much misery on the helpless and infirm, is decidedly convenient.

### THE LEES NURSING HOME, OLDHAM.

A beautiful nursing home, the gift of Mrs. Charles E. Lees, of Werneth Park, was opened last Monday, October 25th, at Oldham. It contains ample accommodation for twenty-two nurses, and great pains has been bestowed upon the lighting, ventilation, and heating of the rooms. One wing, in case of necessity, can be isolated. Every convenience and comfort appear to have been thought of and provided. The work has all been done by local tradespeople, under the supervision of Mr. Tom Taylor. It seems a pity, however, that the laundry in a new home should have been placed in the basement. Mrs. Lees' daughters have furnished the home, and Miss Nicholson, formerly sister at Oldham Infirmary, is appointed matron. A brass tablet to the memory of "Charles Edward Lees, J.P.," has been placed in the vestibule opposite the entrance.

### A VISITING NURSE.

Miss M. P. Thomson, of 63, Drayton Gardens, South Kensington, has announced that she proposes to visit patients of the upper and middle classes as they may require. She is a fully-qualified nurse and masseuse. She holds special certificates for gynæcology, throat and ear work, and doubtless many who cannot afford, or do not require, the constant attendance of a nurse will gladly avail themselves of her services. We wish her all success in her new undertaking.

### A NURSE'S REWARD.

The extent to which people are content to be ignorant of things pertaining to their own advantage is incredible. A case in point occurred lately at Rangoon. Mrs. Simons, the matron of the hospital there, one of the oldest members of the staff, at the age of sixty years sent in her resignation. The committee justly felt that such long service merited recognition, and justly again saw that some provision for her during the rest of her life was the most appropriate way of expressing their regard. Accordingly, the Post Office annuities authorities were asked the price of a pension for her, and they replied that as Mrs. Simons was 60 years of age she was ineligible for one. The committee have, therefore, capitalised nine months' pay for her, which is the highest gratuity they can give. And all the time there is the National Pension Fund, founded and maintained for the benefit of such, and, moreover, granting much more liberal terms to annuitants than the Post Office. But the Indian nurses and hospital authorities are not yet alive to the fact that the nurses working in India have the same privilege in this respect as their English sisters.

### HUCKNALL AND DISTRICT NURSES' HOME.

All classes joined in making a festival of the day on which Lady Belper laid the foundation-stone of the Hucknall and District Nurses' Home. For some time past nurses have been working in the district and winning golden opinions. The association first began with one nurse, soon adding an assistant, and now the new home will provide accommodation for three nurses, a caretaker, and his wife. It is hoped that it will be ready for occupation next spring. The site is in Caddow Park, and is the gift of the Duke of Portland. Patriotic speeches were made by Mr. T. N. Ellis, Mr. J. E. Ellis, M.P., Mr. J. Hind, M.P., and Lord Belper.

Lady Belper, who has taken a great interest in the association, was presented with the silver trowel with which she laid the stone.

### ONE NURSE AND A HALF.

WHAT is to be done in a case where there is too much work for one nurse, and not enough for two? The Board of Guardians at Woburn have been asked to solve the problem by appointing a sewing-woman who will give help in the infirmary. This does not make things easier, as far as can be seen from the outside. Is the newly-employed person to nurse at night and sew in the day? Or is she to sit beside the infirm and ply her needle during the day, whilst the nurse proper sleeps? And why should not the nurse undertake some of the needlework? Could not another nurse, to take alternate night and day duty, have been engaged, both to have undertaken a share of the needlework? The case simply bristles with suggestions, and the one thing impossible is to cut a nurse in two, and employ one half at one place and the other at another.

### DISTRICT NURSING AT SUNDERLAND.

THE district nurse is well established in Sunderland. On the 12th inst. a public meeting in the Victoria Hall was held with a view to affiliating the Sunderland Association with the Queen Victoria Institute. Lady Londonderry made an able and sympathetic speech, and contributed £5 to the scheme. About £2,000 has been raised as a Jubilee thankoffering, which will be invested for the benefit of the association, but it appeared from the proceedings that a few liberal supporters bear the brunt of the expense. Efforts are to be made to get the help of more small subscribers. The number of sick in need of nursing in their own homes is very large.

### STATE AID FOR IRISH NURSES.

THE Irish Workhouse Reform Association is endeavouring to extend a system, already acted on in Scotland, whereby the nurses shall have half their salaries and half their rations provided by the State. As some districts in Ireland are very poor, the boon would be great. In Ireland the salaries of the medical officers of these institutions are already paid in this way, so that the reform would merely extend the principle to the nursing.

### CHARITY ENTERTAINMENT AT HALIFAX.

LARGE and appreciative audiences assembled to witness the dramatic entertainments arranged by Nurse Amy F. Smith in the Mechanics' Hall, Halifax. The first was of selections from "The Yeomen of the Guard," and the second was a comedy, entitled "Married Life." The entertainments were to rouse interest in the West Riding Nurses' Association. Miss Smith founded the association last November with a staff of eight nurses, and already it has been necessary to double that number.

### AN EIGHT HOUR DAY.

THE labour world is fighting hard at the present moment for an eight hour working day. If any one class of workers can be said to be entitled to enjoy such a privilege, none would deny the right to the woman who spends her life in the arduous duty of sick nursing. It is not, however, a question of right, but of practicability. The difficulties in the way are enormous; they may be insuperable. If a rough calculation be made as to the number of extra nurses needed under such a condition, it will be found that from a fourth to a third more must be added to the present staffs, *i.e.*, from 25 to 33 for every 100 already employed. The additional accommodation, food, and salaries represent a very large expenditure; and how is it to be met? Then there are indirect results to consider. These might prove very unpopular amongst nurses. Salaries, for instance, might be cut down, years of training lengthened, probationers provided only with board and residence, as in the Johns Hopkins Hospital, Baltimore; and certainly the competition, even now so keen for good posts, would be sharper still. All good hospital managers are giving the subject close attention, and nurses cannot do better than study the pros and cons very carefully.

### NURSING EDUCATION AT LEWISHAM.

THE Lewisham Infirmary Committee have appointed Dr. Arkell, of Charing Cross Hospital, subject to the approval of the Local Government Board, examiner of probationers prior to their receiving certificates. The examinations are to be held twice yearly.

### HOME FOR INCURABLES, STREATHAM.

A HARVEST festival was held on Sunday last in the chapel of the British Home for Incurables, Streatham, which was prettily and appropriately decorated. There was a large attendance of patients and friends. At the conclusion of an eloquent sermon, Canon Allen Edwards, M.L.S.B., the chaplain to the charity, made an earnest appeal that the east window of the chapel should be filled in with stained glass, not so much as a luxury, but as a necessity, for the present bright light was most trying to the weakened and feeble sight of the inmates. There are fourteen divisions in the window, to fill each of which would cost about £20. The collection for chapel expenses amounted to £4 8s. 6d.

### A LADY DOCTOR FOR LUNATICS.

ONE HUNDRED years ago William Tuke founded the York Retreat in connection with the Society of Friends. He introduced there a humane system of treating the insane, and instituted reforms that have been a model for all following in his footsteps. The recent appointment of Miss Mabel Kemp, a graduate of Glasgow, as junior medical officer is a step quite in accordance with the past traditions of the asylum. Miss Kemp has studied lunacy, and gained experience at Morpeth Asylum.

### COUNTY HOME FOR NURSES AT DORCHESTER.

AN influential meeting, largely attended, was held on the 22nd at the Town Hall at Dorchester to consider the generous offer of the Earl of Ilchester (Lord Lieutenant of the county) to give £500 towards a County Home for Nurses at Dorchester and to subscribe £25 a year. Lord Digby, who was voted to the chair, mentioned that many persons were desirous that the Home about to be started should amalgamate with the County Home for Nurses at Blandford. Resolutions were carried gratefully accepting the Earl of Ilchester's generous help, and to the effect that a provisional committee be appointed to draw up a working scheme for a Home of trained nurses at Dorchester, under the conditions laid down by Lord Ilchester, and also to arrange a plan of amalgamation with the County Home for Nurses at Blandford. It is hoped the results of this meeting will not only largely increase the work of the County association, commenced five years ago, but be of inestimable value to the rich, to the middle classes (so often forgotten), and to the poor.

### Lectures to Surgical Nurses.

By H. A. Latimer, M.D. (Dunelm), M.R.C.S. of Eng., L.S.A. of London, Consulting Surgeon, Swansea Hospital; President of the Swansea Medical Society; Lecturer and Examiner of the St. John Ambulance Association, &c.

### XVII.—REGULATION OF VISITORS—ATTENDANCE ON INFECTIOUS CASES—DIETING AND WASHING PATIENTS—NURSES' TREATMENT OF HEADACHE AND DELIRIUM.

In continuation of what I was saying in my last lecture on the subject of nursing patients in private houses, in all illnesses you will have trouble about the visitors who wish to see your patients. Take the directions as to what shall be done in this matter from the medical attendant and abide faithfully by the same. But I must tell you that in no part of your work is so much tact required as in this, for people are very prone to take offence, in private nursing, by being excluded from the sick-room. If you explain that you have been told by the doctor to act in such and such a way, as the case may be, the friends take the matter sensibly and you escape from being blamed. It is a good plan not to allow more than one, or at the most two, people in the room at the same time, and to see that they do not stay long enough to fatigue the invalid, and that their conversation does not run on other people's illnesses. Sick people are peculiarly fond of fancying themselves to be suffering from symptoms which are reported to them as affecting other people, and to be depressed in consequence.

If the case which you are nursing should be of an infectious nature, careful isolation from the healthy folk in the house should be maintained. Have a large sheet nailed over the outer side of the door of entrance into the room, and let this be kept wet with some antiseptic solution; by adopting this measure you will prevent the escape of floating germs from the sick chamber. Mix disinfectants with all discharges from the patient before you carry them away, and empty all such things yourself, and do not trust them to servants, who are liable to leave them about until they find it to be convenient to attend to this matter. Use antiseptics freely about your own hands, and when you go out for exercise wear a different dress to the one you have had on when in attendance upon the invalid.

The dieting of the patient next calls for remark. In all cases you will receive instructions from the surgeon in attendance as to the special diet which is required. For many years past the plan of lowering diet in fevers has been abandoned, and with the happiest results. Now we feed and stimulate our patients when suffering in this way, but we are careful to give them foods which require but little effort of digestion; for, during the feverish state, the digestive organs are working faultily, and unless one is careful on this point much harm is done. Of all foods given in this condition, none exceed in value milk, for this contains all the flesh-forming principles; at least three pints a day of this fluid is required for the support of an adult person. Mixed with soda water, it is, as a rule, very readily taken by feverish people, for it then has a very refreshing effect. The various animal broths are also given as alternative foods. Of them, at least 1 lb. of beef, mutton, or chicken, prepared as broths, should be administered in 24 hours, and, with them some variety of farinaceous food should be combined. Some kind of peptonised food is very likely to be ordered, or one or more of the prepared beef-jellies or beef-juices which are in the market. The patient's appetites will be bad, and in many cases their repugnance to taking foods and medicines will be very extreme. If you attend carefully to the condition of the mouth and tongue you will reduce these difficulties to a minimum. If the patient only complains of this want of appetite, and has, as is usually the case, a furred, dirty tongue, he will be much comforted by rinsing the mouth out with hot water in which a little borax has been dissolved; and if the tongue should be very dry and the teeth encrusted with mucus, much benefit will result from your anointing the former with pure salad oil, or with glycerine and water, or some form of jelly, and in cleansing the latter with a small mop of clean lint wrapped and tied round the end of a piece of stick and charged with some toilet vinegar and water or some mild antiseptic lotion. You will be surprised to see how much benefit will result to your patients from such attentions as these.

You will have been fully taught how to wash invalids whilst you are working in our hospital as probationers, so I will not dwell upon the methods you are to use in carrying out this most necessary part of nursing—most necessary because the skin is an important organ for carrying out of the system poisonous matters, and for reducing the temperature by means of its natural function of perspiration. Add to these facts that it affords a large surface in which blood-vessels lie, and that by cold applications to this surface the general warmth of the blood can be reduced by the blood which has been chilled in these vessels being carried away again to circulate in the interior of the body, and you can readily perceive what a valuable thing cold bathing is in fevers for the general reduction of temperature. When this has risen above a safe point, and has amounted to that degree which I have spoken of as constituting hyperpyrexia, the only measure which has been found successful in many cases in reducing the heat to a degree consistent with safety to life has been the one of wrapping the whole body in wet sheets which have been wrung out of tepid or cold water, or in immersing the patient in one or more baths of warm water, which is cooled down to 70 deg., 60 deg., or even 50 deg. Fah. (Whitla), whilst he is in it; and in lesser degrees of heat than the one I have just alluded to you will often be directed to sponge limbs with cold water, or to apply metallic coils of tubing which allow of the circulation of cold water within them, and which, being appropriately shaped for the head or limb to which they are applied, cool that part which they encircle.

Now I will tell you how to deal with some of the "complications" which arise from time to time in fevers. A greater or lesser degree of headache generally prevails. The milder forms are best treated by your applying some spirit lotion to the forehead—such as eau de Cologne and water—and by your combing and brushing the hair; you will be surprised oftentimes to see how much comfort follows the latter proceeding. If the hair should be long, as in the case of women, and the fever should be one which is likely to continue for some time, it will be well to have it cut short. By doing this the patient will be much benefited, as she will be saved the long and tedious combing which must be done often to keep things tidy, and her hair will be strengthened. She will have to lose the greater part of it, whatever is done, for the hair is shed—to be replaced again by a new growth—after fevers. A greater disturbance in the brain than what results in headache leads to delirium, or to unconsciousness or coma. You may often tell when the latter is coming on by seeing the patient looking wildly about him, or staring with a fixed attention at some object in the room—a picture, a screen, or what not. It rarely happens that you cannot succeed in quietening the sufferer by speaking to him in a firm way, and so, by recalling him, as is the figure of speech, "to himself." True, he will relapse and talk again, but the same method of dealing with him can be again adopted. If he is suffering from illusions, and thinks that someone is lurking behind the screen at which he is gazing, the screen can be moved, and then oftentimes the diseased fancy will be removed as well. If the delirium should be of an active and effective character and should lead to his attempting to jump out of bed or to commit violence, two or more people will be necessary as attendants; and from them more good will be done by loud peremptory orders, issued as occasion demands, than by manual restraint, though such restraint may be called for at times. It is not often that you will have to deal with this violent form of delirium in fever cases, and, as a rule, constant watchfulness and persuasion will suffice to deal with those you have under your care.

### The Management of Tracheotomy Cases.

By C. F. MARSHALL, M.D., F.R.C.S., Late Registrar to the Children's Hospital, Great Ormond Street.

TRACHEOTOMY cases are the most anxious ones that the nurse is ever called upon to deal with. It is first advisable to say a few words concerning the different kinds of laryngitis we meet with, and the cases in which tracheotomy is or is not required. Without a proper knowledge of these cases it is often difficult for the nurse to understand why the surgeon is in a great hurry to perform tracheotomy in some cases while in others he puts off the operation as long as he can.

Tracheotomy is performed to save a patient from impending death under the following conditions: (1) A foreign body lodged in the larynx, trachea, or bronchus; (2) a scald of the larynx; (3) obstruction of the larynx from acute inflammation, usually due to diphtheria, occasionally to simple laryngitis; (4) obstruction due to chronic inflammation, usually tuberculous or syphilitic; (5) obstruction due to new growth, such as cancer. Let us take these in order.

1. A foreign body, such as a nutshell, a farthing, or any small body may become lodged in the larynx, usually in children. If this is not soon coughed up, and if it cannot be extracted by forceps, it is necessary to perform tracheotomy and try and extract the object. If it has descended into one of the bronchi the tracheotomy wound must be held open with tapes till the object is coughed up. No tube should be used in these cases.

2. A scald of the larynx is usually caused by a child applying its mouth to the spout of a kettle and inhaling the hot steam. Acute œdematous swelling of the larynx is thus caused, which may require tracheotomy to avoid suffocation.

3. By far the majority of cases requiring tracheotomy are those of diphtheria affecting the larynx and trachea either primarily or secondarily. In these cases the obstruction is due to the thick yellowish-white membrane which is formed on the mucous membrane of the larynx and trachea, and often in the bronchial tubes as well. In most cases of diphtheria, especially in adults, the larynx is not affected; but in children it is very common for the disease to spread to the air passages from the throat, and in some cases for it to originate there. In the latter case it is often very difficult to diagnose whether the case is one of diphtheria or of simple laryngitis unless pieces of membrane are actually coughed up. Acute inflammation of the larynx often occurs at the onset of acute fevers in children, especially in measles and whooping-cough, and may be so severe as to suggest diphtheria; in fact, if no membrane is seen in the throat it is often difficult to tell at first the nature of the case. The signs of laryngeal obstruction are the same in both cases, viz., the laboured breathing and sinking in of the ribs and sternum during every inspiration (known as "recession"), the hoarse, "brassy" cough, and the loss of voice. The amount of laryngeal obstruction is known by the degree of recession of the ribs and sternum, but this is no guide in diagnosing between simple and diphtheritic laryngitis; in fact, it is often so excessive in patients with acute laryngitis occurring in measles as to appear to indicate immediate tracheotomy, yet such cases nearly always get well without it. A better indication is the general condition of the patient. In simple laryngitis the colour of the face is usually good, even if a trifle blue; the pulse is usually full and strong; the temperature generally rises rapidly to 103 degrees or more; the onset is as a rule very sudden; albumen in the urine is not common. In diphtheria the face soon becomes of a dull "earthy" colour; the pulse is rapid and soon becomes small; the temperature usually is not so high, but varies much in different cases; there is usually albumen. As most of the simple cases are due to measles or whooping cough, it is of course important to look out for other signs of these diseases, viz., the appearance of the rash and coryza in the former, and the swollen eyelids and characteristic cough in the latter. These further signs usually appear in a few days after the laryngitis, and when seen clear up the diagnosis. As regards tracheotomy in such cases, the best rule is to do it early if the diagnosis of diphtheria is certain; if doubtful, postpone it till the case becomes urgent. If the probability is in favour of a simple laryngitis, tracheotomy should not be done if it can possibly be avoided, (1) because such cases nearly all get well with ordinary measures; and (2) because if it is done broncho-pneumonia is likely to follow. A few cases, however, become so urgent as to require operation. There is another form of laryngitis occurring towards the end of fevers, especially in measles, known as "membranous laryngitis." This is characterised by the formation of membrane the same as in diphtheria. It is, in fact, probably the same disease, and at any rate for all practical purposes must be treated as such.

4 and 5. Tracheotomy for chronic obstruction due to tubercle or syphilis of the larynx is uncommon. In cancer it is done as a preliminary to some operation for removal of the growth.

### THE OPERATION.

As the operation is often done on an emergency, when little assistance is at hand, it is necessary for the nurse to know what to do in order to assist the surgeon when no other doctor is present. On such an occasion the surgeon gives enough chloroform to last during the operation as a rule, if more is required the nurse must continue giving it. The chief thing for the nurse to do under these conditions is to hold the patient's head firmly between her two hands, so that the neck is slightly stretched and the head somewhat bent back over a small pillow or folded towel placed behind the neck and shoulders. She must see that the head is kept perfectly straight, so that the nose and chin are in a line with the middle of the body. When the trachea has been exposed, her next duty is to hold the retractors one in each hand to hold the wound open, at the same time holding the head steady between her forearms. Some surgeons, however, dispense with retractors.

### MANAGEMENT OF THE TUBE.

The best form of tube, and that now in general use, is Parker's. This is a silver tube, consisting of three distinct

FIG. 1.—Parker's Tube.

parts: an outer tube, an inner tube, and a moveable shield. The inner tube projects a little beyond the end of the outer one so as to clear away any mucus adhering to the latter. The duty of the nurse is to remove the inner tube occasionally to clean it in a solution of hot soda; the less often this is done the better so as to disturb the patient as little as possible. On no account should the nurse remove the outer tube; if there is any occasion to do this it must be done by the surgeon, as there is sometimes difficulty in

Fig. 2.—Tracheal Dilator.

replacing it. The illustration (Fig. 1) shows Parker's tube complete and also the inner tube separate. Sometimes the patient, if a young child, pulls the whole tube out; the nurse must then immediately insert the dilators and send for the surgeon, she should not attempt to replace the tube except under circumstances where no surgeon is available for some time. As long as the dilators are held in the trachea there is no danger of suffocation. In order to avoid this accident it is usual to tie the child's hands to the side of the cot; in older children this is not necessary.

With regard to covering the tube with warm sponges or gauze pads I think more harm than good is done by them, for unless they are constantly changed they become cold and clammy. If the temperature of the room is kept at 70 deg., and there are no draughts, such things are unnecessary.

*(To be continued.)*

### Typhoid Fever at Maidstone.

### EMERGENCY HOSPITALS.

The first of the eight emergency hospitals is close to the L. C. and D. station, and the roadway in front of it and of some of the others is thickly strewn with tan to deaden the sound of the traffic. There is no outward sign of a hospital, unless a nurse passing quickly across an upstairs window can be taken as such. Two shops, with the floors over them, and the large hall of the Salvation Army, are the component parts of what is commonly spoken of as "The Station Hospital." The shop windows are painted over half way up to protect the patients from the inquisitive espionage of the local juveniles, who now flatten their noses in vain against the glass. They find with disgust that the reflection of their own little faces is all that the windows can offer them. The shops and the rooms above have all been converted into wards, and very workmanlike they look. When the patients were first carried in they were all serious cases, and many were delirious. They were placed in charge of trained day and night nurses, and now their appearance and condition do credit to doctors and nurses. A temporary kitchen has been erected between these houses and the Salvation Hall, and the latter has been converted into two fine wards, one above the other. About 80 patients can be accommodated in the Station Hospital, men, women, and children having their respective wards.

The superintendent of the emergency nurses has her headquarters at the Station Hospital and visits all the others in the course of the day. It is only a short walk to the Hedley Street Mission Room, which has been turned into a very neat ward for men and boys, some of them very bad cases. At the end of this temporary ward a smaller room has been turned into a convenient kitchen, and a small plot of garden ground at the back is also utilised. There is a water-cart outside this little hospital, for unfortunately the supply throughout the town has been inconveniently precarious, and sometimes altogether absent. To reach Perry Street, Perryside, the route lies through some very poor streets. The temporary hospital there contains a row of beds on either side of its wards and cots down the middle, all occupied.

Padsole Hospital is nearer the centre of the town, and consists of two wards, one above the other. All these four emergency hospitals stand on the same side of the river, the others being over the bridge and some distance away.

There is one block at the permanent Fever Hospital where two wards are given over to the typhoid cases. They are bright, airy rooms, and the view from the windows is lovely. The Bournemouth tents have been pitched near by, and form very convenient little wards.

The Wesleyan Schools on the Tonbridge Road consist of two rooms adjoining, the larger having a partition down the middle and a double row of beds on either side of it. The Congregational Schools at Westborough now form a single ward, which is lofty and airy.

The Milton Street Mission-room has from the first received an exceptional number of bad cases, transferred there from houses in the neighbourhood, which seems a very poor one. It consists of a single room, with kitchen, &c.

All the emergency hospitals have a smart, orderly appearance, and gay autumn flowers give a touch of brightness to the whole.

The first cases of typhoid were placed in the West Kent Hospital, which soon gave up four wards for their reception, fifty patients being admitted there.

In passing from one to the other of these emergency hospitals it is evident that the normal condition of the town is unchanged. The inhabitants who have escaped the disease pass on and attend to their ordinary avocations, wondering a little sadly why their pretty and afflicted little town should now be described as "deserted, shunned, and panic-stricken," when, save for the presence of extra doctors and many nurses, so little change is outwardly to be seen.

### The Meath Home for Epileptics.

### Presentation to the Matron.

We regret to report that Miss Mabel Anderson, for five years lady superintendent of the Meath Home for Epileptics, Godalming, has had to resign her post, the doctor ordering her at least six months' complete rest, when we hope she may be able to take up other reins again elsewhere. Miss Anderson's work at Godalming has been quite unique, and she leaves a complete organised scheme, every detail of which has been thought out by herself. The home is always quite full, there being over seventy epileptic women and children, besides nurses and staff, and during the five years the house has been twice enlarged and the chapel built. The committee presented Miss Anderson with a beautiful gold, large heart-shaped locket and chain, containing a picture of the home she loves so well, and the following inscription: "Presented to Miss Anderson by the Committee of the Meath Home of Comfort, in memory of her devoted service as Lady Superintendent, 1892-97." The nurses, patients, and staff gave her a fitted-up dressing bag of brown morocco leather, fittings of ebony and silver, with monogram on each article, and on it, "Presented to Miss Mabel Anderson, our matron, by the staff and patients of the Meath Home, as a mark of love, and regret at her leaving them. September, 1897." Mrs. Cousins, who has worked in Stepney, is appointed matron of the Meath Home.

### Notes on the Plague.

By a Nurse in the Plague Camp, Kirkee, near Poona.

The bubo of plague appears in the axilla, neck, groin, and abdomen. That in the abdomen is most rare, and is rapidly fatal. It is accompanied by intense abdominal pain, eyes starting from the sockets, continuous vomiting, and extreme restlessness. The patient is difficult to feed from the onset, and no drugs seem to give relief.

In disguised cases the temperature becomes suddenly high, great pain is experienced all over the body, vomiting sets in, the patient refuses all food, has no sleep, mutters low in delirium, there is hæmorrhage from the throat, lungs, bowels, &c., and this is quickly followed by death.

The eyes are congested, the tongue furred, and sometimes there are characteristic pimples on it.

Unconsciousness is nearly always present to a greater or less extent, and the whole body is subject to a peculiar, continuous tremor. Danger arises from heart failure when the temperature begins to fall. I have never known a case of pneumonic plague recover.

The nurse, in wiping the expectoration from the unconscious patient's lips, must use lint dipped into a disinfectant, such as Condy's fluid. She must also be careful not to inhale his breath, which is usually very offensive.

Case I. (Recovery), April, 1897.—Patient, about 25 years of age, rather thin than otherwise, was admitted about three hours after appearance of bubo in the right groin (as far as he knew); very excited and trembly; said he had not felt quite well for three days previously. On admittance temperature 102·6 deg., rising to 106, in evening; slept fairly well. Second morning—temperature 98; noon, 99; four p.m., 102 6; half-past seven p.m., 103·4. Had been in high spirits all day, when he complained of great headache and depression; hands trembled so much that he could not hold anything; cut hair close to head, and applied iced evaporating lotion day and night; very restless during night in spite of draught. Third morning—vomiting very troublesome; gave bismuth, and applied mustard plaster over pit of stomach. Not really delirious, but subject to strange fancies; feeds of milk, beef-tea, and chicken soup, in quantity ℥ij, iced; brandy every third hour, ʒiii.; great thirst, gave ice to suck, and small iced soda drinks; pulse weak and high; complained of great depression on chest; tongue covered with thick white fur. After this the temperature was 104·2 (ranging from 103·4 for four days). Morphia injections for five nights; slept little; difficult to get to take his food. Eighth morning—temperature 102 (coming down on ninth morning to 99, and on tenth to 96·8). From eighth day brandy increased; patient inclined to be hysterical. From fourteenth day patient much better, but nerves completely wrecked. Discharged from hospital in a month's time; had complete rest and change. In this case the bowels were very constipated in beginning of illness, with great abdominal swelling. Gave turpentine enema, simple enema, and calomel at different times. The bubo, which was very tense and painful, was painted with belladonna for the first three days; then, as it did not disperse, an incision was made and poultice sprinkled with iodoform applied. A small quantity of sticky fluid exuded from the wound; then, as it healed too rapidly, the wound was probed, and eventually a larger incision was made. A slough formed, which was touched with blue-stone, after which it healed rapidly.

Diet.—Milk and lime-water, chicken soup, beef-tea, all iced, for the first six days, in quantities not exceeding an ounce and a half at a time. After fourteenth day took chicken diet with extras; patient seems in perfect health.

Case II. (Fatal), April, 1897.—The patient was a man of about 50 years of age, very heavily built, with goître on each side of the neck, and looked a fit subject for fatty degeneration of the heart. He was brought into hospital on the third morning of the fever unconscious, sleepless, and very restless indeed. Temperature 105; expectoration thick and sticky, in great quantities; diarrhœa violent and continuous (had had aperient before leaving home), tongue characteristic, and eyes congested. Fourth morning—temperature 104 deg., falling to 103 in evening; speech no longer clear. Fifth morning—temperature, 101 deg., rose to 102 deg. at noon; restlessness terrible; unconscious; had ether and digitalis injected into region of heart twice; large mustard plasters; died at half-past four p.m. of heart failure. On admittance there was a decided thickening of the left groin. It was a distinct bubo. Death very sudden.

Case III. (Fatal), May 14th, 1897.—Patient (very bad subject) elderly man, 77 years of age, no teeth, defective sight and hearing; for years had suffered from paralysis of hands; admitted on third morning of fever. Fourth morning—temperature 103·6, rising to 104 by evening, pulse in right hand imperceptible, in left so intermittent that it could not be counted; sleepless, talkative, occasionally wandering, expectoration thick but not frequent, very restless, impossible to lay on either side. Had draught at night, but slept only one hour; very delirious. Fifth morning—passed no urine all night; fomented over bladder, no result; catheter passed, but only a few drachms were drawn; salt fomentations over kidneys, digitalis poultices; temperature 103, rising to 104 at seven p.m.; low muttering delirium, respiration very hurried; abdomen seemed full of fluid. Brandy given frequently in doses of ten minims. Spits out nourishment sometimes. At half-past five p.m. ether and digitalis injected into region of the heart; digitalis poultices applied to the loins; cold perspiration. Died at twenty-five minutes past nine p.m. About three minutes before death there was a flow of reddish fluid from the mouth and a general convulsion of the body. Bubo, very hard to the touch, appeared in right groin on third evening of fever, was leeched, painted with belladonna, and hot salt fomentations applied. Temperature was kept down for first two days by doses of soda salicyl. Digitalis, ether, ammonia, carb. and sp. ammonia aromat. were given freely.

### Women as Hospital Dispensers.

There can be no doubt that the example set by many of the children's hospitals might be followed with advantage by the general hospitals too, and that the employment of properly trained and competent women as dispensers would be a wise step to take in many institutions. There can be no doubt that dispensing offers a very useful opening for women workers. Mrs. Swain did good service by training women in dispensing, a work which is now continued with success by Miss Bradbury, resident dispenser at the Ryde Dispensary, Isle of Wight. We have been asked how many women are certificated dispensers, and whether it would be possible to provide a senior and say half-a-dozen junior dispensers, all women, and all fully competent to undertake the work of a large dispensary. The Editor would therefore be glad if those ladies who are trained dispensers in active work would send him particulars of their experience and training, and the emolument which they would expect to receive. All communications should have the word "Dispenser" written on the outside of the envelope, and it would be useful if replies stated the age of each correspondent. We hope that, providing those who are now qualified will declare themselves, it may be possible to improve the prospects of women dispensers for public institutions.

### A Book and its Story.

### MOLIÈRE AND HIS MEDICAL ASSOCIATION.

THE wit of Molière forms a part of the common gaiety of nations. Every schoolboy who has reached his teens has either laughed or trembled, perhaps the latter more frequently, when standing up to construe into bald and broken English the polished French in which are set forth the drolleries of the "Médecin Malgré lui" or the "Malade Imaginaire." "Doubtless," says the author of the work* before us, "the medical absurdities of which Molière was witness, sufficiently explain the manner of his ridicule. As a satirist more than as a comedian, he laughed at their pretensions, but the causes of the persistence and severity of his railleries remain obscure." To elucidate this enigma is the object of Dr. Brown's monograph; and, although he may not have succeeded in this object, he has brought to the task which he has undertaken a sufficient literary capacity and, what is even more important, a very intimate knowledge of the topics with which he deals.

Jean Baptiste Poquelin, better known to fame as Molière, was born at Paris in the year 1622, and was called to the Bar, but he never pleaded as an advocate. The attractions of the comic stage were irresistible. "At the age of 22 he joined the Illustre Théâtre of the Porte de Nesle, and struck into the rugged path that leads to fame." Of his first stumblings along that rugged path, the hill difficulty, on which so many pilgrims of literature lose heart and turn aside, few records seem to remain; but it is known that from the first he loved the Society of the Médecins Ambulants, the quacks who strolled about catering for practice by the sale of love-philtres, magic pills, plasters that would cure anything, and whose patients were found in the ignorant audiences, who had no confidence in the regular practitioners of the day. From these he drew the earliest of his medical impressions. But it is not to be doubted that he studied character among the legitimate professors of the healing art. Dr. Brown points out that Molière's company performed near Montpellier, where the medical faculty was the rival of the faculty of Paris. An account of their quaint method of creating a doctor of medicine is preserved by the English philosopher, Locke. "A grand procession of doctors," says Locke, "in red and black caps, ten violins playing the airs of Lulli. Then the president takes his seat; the violins cease, and he begins to speak; eulogising his colleagues, he forthwith delivers a diatribe against innovations and the circulation of the blood. The recipient in turn compliments the Chancellor, the Professor, the Academy. Again the violins; and the president takes a cap, which an attendant had been holding upon the point of a staff throughout the procession, places it upon the head of the newly-created doctor, puts a ring on his finger, and then passing a gold chain round his waist, prays him to be seated."

At the age of 36 Molière returns to Paris, bringing with him a vast quantity of theatrical pieces which he had performed in the provinces; and amongst these were "Les Trois Docteurs Amoreux," "Les Trois Docteurs Riveux," "Le Médecin Volant," "Les Fagoleux," "Le Médecin par Force," &c. "So fair a list," says Dr. Brown, "with medecine for their theme, is, to say the least, significant, and shows that from the first the subject had for him a certain attraction that should be borne in mind." At the performance of his plays the patronage of the Court was, however, extended to him, and we next find him mingling, as a Royal favourite, in the brilliant crowd which clustered round the King. The character of the Court seems to have placed the medical man of that day in a difficult position. "The career of medicine," Dr. Brown explains, "offered only two alternatives—that of cultivating general practice, associating with equals, letting the world take its course, and quietly laughing at those who lead it; or that of engaging in the service of His Majesty or some noble at the sacrifice of liberty and independence—a post that few could possibly obtain." It is certain that between the two classes of doctors there must have been a ceaseless warfare, and it is needless to say that the doctors differed even within the precincts of the Court, and Dr. Brown gives many examples of their strange behaviour on such occasions.

Louis Quatorze had, before he was 20, suffered from a variety of ailments, of which a list, almost too loathsome to be printed in a medical journal, will be found at page 72 of the volume before us; and by his command there was kept "Le Journal de la Santé du Roi," which contained a record of these ailments and the manner in which they were treated. This volume, hitherto in MS., is preserved in the National Library of France, where it has frequently been examined by historians. It reveals the pedantry, the ignorance, the folly of the doctors, whom Molière, drawing their characters with scarcely an exaggeration, caricatured in "L'Amour Médecin." "All Paris," we are told, "runs in crowds to see the Court physicians represented on the stage. . . . Such is the way they ridicule those who kill people with impunity."

But we are in danger of exceeding the space at our disposal and must hasten to a close, though there is a great deal in these fascinating pages to which we should have liked to call attention. The circumstances which led Molière to write the immortal "Misanthrope," "admittedly a reflex of his own case," says Dr. Brown, will be found at pages 103 to 111. This section of the work is followed by an admirable summary of the "Médecin Malgré Lui," in which Leandre, who pretends to be an apothecary, is astounded to hear Sganarelle assure him that all he needs to do, in order to carry out the deception, is to don the dress of a learned man. It is not even necessary to know any medical terms. He himself knows nothing. "I am run after on all hands," says the famous impostor, "and if matters go on as they are I've a good mind to remain a doctor all my days. It is the very best of trades, for whether you do right or wrong you are paid all the same. . . . Lastly, the great advantage of the medical profession is that the dead are the most discreet and honest people possible to deal with in the world. They are never heard to complain of the doctor who sends them out of it." As the author shrewdly observes, true satire could scarcely go further than this.

The last years of Molière were darkened by those domestic sorrows over which he broods in the "Misanthrope." "Great imaginative and artistic powers," says Dr. Brown, "seem almost incompatible with the ordinary normal standard of humanity. Where such qualities exist, the intellectual balance is unequal, and the characters unsuited for the parts we have to play in common life. Molière is an illustration of the fact." In addition to these defects of character, the great comedian was constantly ill, and the end came in February, 1673, when, during a performance of the "Malade Imaginaire," he was struck by a fatal illness, and died within an hour of leaving the stage. We have read this little volume with unfeigned enjoyment, and close it with reluctance; but we are bound to say that, in our opinion, Dr. Brown comes to the last page of his attractive work without having solved the problem which he set himself when he took up his pen—what was the real cause of Molière's hostility to the medical profession? Perhaps, after all, the answer is to be found in a suggestion made by the author himself, that as Molière drew his wit and portraiture from whatever source lay open to him, the doctors were the order destined to receive the buffets of his immortal critique.

* "Molière and his Medical Association." A. M. Brown, M.D. (London: The Cotton Press. 1897.)

### A Nurse's Visit to Texas.

### VI.—HOME AGAIN.

TRAVELLING and camping out in bad weather are quite different matters, as I found when I went with my brother to Austin, thirty-five miles, in January to fetch stores and fencing wire. We had had a good deal of rain that month, and on the return journey, which took two days, the roads were so bad that heavy wagons kept sticking, and twice the first day we had to lend our horses to help drag one out of the mud. It was so deep that when we halted I took an axe and cut it away from between the wheel-spokes in solid blocks reaching from axle to tyre.

We only travelled nine miles that day; at sunset camped in a cedar grove, and that night we had the most tremendous thunderstorm I ever experienced. The rain began just as we finished our supper, so we packed everything into the wagon, piled the sacks of flour, salt, and sugar in the middle, and covered them with rugs to keep them dry; then we lay down on either side of the heap, talked, and listened to the storm. Every now and then the rain would begin dripping through the wagon tilt, and we had to get up and draw it tighter. I have slept on odd beds before and since, but that night's was the strangest I ever had—a Gladstone bag and a coil of fencing wire, while my brother lay on the horses' harness; yet after the thunder stopped we both managed to sleep. I enjoyed that journey, I suppose because it was a novel experience.

I stayed in Texas through the spring, which was delightful; the pastures were covered with flowers, and the brush coming into leaf showed every tint of delicate green and brown, with here and there a purple judas tree, which, like our blackthorn, blossoms before its leaves come out.

I began my return journey early in May, and travelled by rail from Austin to New York instead of taking steamer from Galveston, because small-pox was still about in the southern towns, and my brother thought it safer for me not to go through the seaport.

The railway journey took 65 hours, but it was very enjoyable; my fellow passengers were most kind, and a woman travelling alone in America is so well looked after that difficulties are almost impossible. I reached New York on Sunday evening, secured my berth on the ss. *Britannic* to sail on Wednesday, stayed until then at Taylor's Hotel, Jersey City, and spent the intervening days in exploring New York. I had a calm return passage and such pleasant fellow travellers that I felt quite sorry to find myself in the Mersey once more, and to think that my American trip, which had quite restored me to health and given me so many new experiences, was really over.

Looking back on those months in Texas, the life seems exceedingly pleasant, but then I was only a visitor and had not time to feel the monotony of it; I was told that after a few years nearly all the educated young men who go out throw up ranching and drift back to the towns, trying to get work there, with very little success. There are no fortunes made over ranching now, and the young emigrants are disappointed when they find, instead of the Texas of their dreams, a hard life, pleasures few and simple, and no prospect of making more than a bare livelihood. If young men would only work as hard and live as plainly at home as they are forced to do on their ranches, they would stand more chance of getting on here than they do there, where they live the life of English farm labourers and are isolated from their friends.

When it is a question of health it is a different matter. In spite of great and sudden changes of temperature, the climate of Texas in the hilly districts certainly benefits consumptives from the northern States and Europe. But if the invalid goes out with the intention of working and not merely getting well, he would be wiser to try to hire himself to work on someone else's ranch rather than invest his money in buying one of his own, at any rate until he has been out long enough to know something of the ways of the country, for times are bad in Texas, and there does not seem to be much prospect of their improving just yet.

The State was more flourishing in the times of slavery: when that was abolished cultivation on a large scale with free labourers no longer paid. After that the plantation days were over; so are the days of the big cattle ranchers, who were not very particular whether the cattle they collected in the round-ups bore their own brand or their neighbours'. Now that there is more law and order in the State, and everyone has to hand over a bill of sale to the purchaser with the cattle or horses he is selling him, stating that he himself came by them honestly, all that is impossible. Horse stealers are more severely dealt with than murderers, and property is as secure and life as tame and free from adventure as in out-of-the-way rural places in the old country.

### Everybody's Opinion.

[Correspondence on all subjects is invited, but we cannot in any way be responsible for the opinions expressed by our correspondents. No communication can be entertained if the name and address of the correspondent is not given, or unless one side of the paper only is written on.]

### THE PENSION FUND.

"A GRATEFUL NURSE" writes: I beg to acknowledge receipt of a copy of THE HOSPITAL. I have been a constant reader of that very valuable paper ever since its commencement, and have got from time to time a great deal of information and many useful hints from its pages. I am a Pension Fund nurse, and will take this opportunity of thanking you and your colleagues in the council for all the interest you have taken in our welfare. I feel extremely grateful to all the gentlemen who have given money, time, and talent to our fund. I hope nurses will not be led away by the new scheme that has been set afloat lately. I shall stick to the Pension Fund myself. I put nearly twenty years' savings into it about seven years ago, and do not regret having done so. If you think this letter worth publishing in THE HOSPITAL, please do so. I wish your paper and our fund much success.

### THE ROYAL BRITISH NURSES' ASSOCIATION.

"NURSE H." writes: Knowing the kindly interest you take in nurses and all nursing matters, I cannot help asking your advice in the question of the Royal British Nurses' Association. Much to my surprise, I have this evening received the enclosed papers, and I am puzzled to know how to act for the best. It is mean to resign membership just at present. Is this meant as an insult to our esteemed President?

*** We do not think it fit to advise nurses as to their conduct towards any institution, but we would point out to our correspondent that the whole aim of the circular which she encloses, sent out by the so-called "Members' Rights Defence Committee" (who are still anonymous), is to prevent the adoption of the revised bye-laws of the Royal British Nurses' Association—revised under the direction of a special committee and with the approval of the honorary officers, and we think we may unhesitatingly say with the approval of the Royal President also, since so important a step would not have been taken, we imagine, without consulting her wishes. Therefore it would appear that what the small faction who sign the circular sent to the members of the Royal British Nurses' Association are trying to induce them to do, is practically to pass a vote of censure against their President, their honorary officers, and their council. It is for nurses themselves to decide whether such a step is desirable or fitting. The present question before the nurses of the Association is, in a nutshell, simply this: In whom have they most confidence—in their Royal President and their honorary officers or in Dr. and Mrs. Bedford Fenwick.—ED. *T.H.*

### The Victoria Commemoration Club.

THE Secretary of the Victoria Commemoration Club prepared a charming "At Home" for the members and their friends at their premises, THE HOSPITAL Building, Southampton Street, Strand. The rooms were elegantly arranged and adorned with flowers. In the drawing-room a selection of excellent music, given by professional and amateur artistes, proved so attractive that additional chairs had to be brought in. Miss Maude Danks sang "Sing, ye Birds" and that unreasonable but pathetic and favourite ballad "Douglas Gordon," and Mr. Kendle Ward "By the River" and "Come to Me." Mr. Alec van Homrigh created great amusement with his two well-chosen and humorous ditties, and Miss Margaret Turton, looking very pretty, recited with much dramatic fire "The False Light of Rosilly" and "The Ladies of St. James." Tea, coffee, and an abundance of cakes and sweetmeats of all kinds were most attractively served in the dining-room, to which, for the occasion, the writing-room was added. The majority of members have not yet grasped the extent of the privileges and freedom offered them by the club, for they have not yet learnt to feel quite at home in it. This shyness will undoubtedly wear off as they realise that it is their own club, and that all the delightful advantages and conveniences have been arranged to meet their requirements. All that remains for them to do is to show their appreciation by using them freely.

### Sensitiveness.

BY A MATRON.

THERE are two forms of sensitiveness; the one provokes pity, the other calls forth scorn. Thin-skinned people, as we know, fare badly when left to the mercy of their fellow creatures. They invite by their very manner and appearance ridicule and chaff, and court the pointed sarcasms and withering remarks which help to make some lives miserable. They shrink into the mysterious recesses of their inner selves at small provocation, and retire worsted in case of open warfare of tongues. They suffer in silence but acutely, and seem badly adapted to meet the jostles and rubs of the world's rabble. Nor in the narrower sphere of the nursing world is the crowd more mannerly—nay, perhaps, from the very limitations of its sex, it is less benevolent and more inclined to hustle the timid. Yet it is often in sensitive natures that we find the most sympathy, and a good nurse must be sympathetic. Sick folk demand it, they require those about them whose feelings are easily touched, whose compassions flow forth spontaneously. Therefore we cannot exclude from our ranks those who are not gifted with a pachydermatous hide, though for their own sakes it may be well for them to cultivate one.

But there is a point where sensitiveness ceases to be a folly and becomes a fault. When it consists wholly of self-consciousness it is insupportable. When every detail of social and professional intercourse is twisted and perverted and made into a personal matter, and the mind becomes concentrated entirely upon itself, it is a condition which calls for reproach rather than sympathy. It is pitiful to find how far-reaching are the limits of this egotistical spirit, and would sometimes be incredible if not known to be actually true. Only those who have dwelt in a colony of women divided amongst themselves could believe the tiny atoms of indiscretion which are construed into grave offences, the paltry trifles which assume gigantic proportions, the merest sparks that blaze into unquenchable flames all because self-pride has been wounded, self-satisfaction ruffled, and dignity overlooked. And feminine methods of retaliation sometimes nearly approach the feline.

Why is it that when full-grown and responsible women have taken the yoke upon their necks and decided to give themselves to "the duteous life which looks not forth beyond its narrow sphere, and finds its work, and works it out," that they should spoil their whole ideal by introducing this spirit of morbid sensitiveness? It is so incongruous, like cloth of gold embroidered with wool, or a green field studded with advertisements of Beecham's pills. Why have I been overlooked? Will the blame fall upon me? I must exonerate myself in case I am accused, and so forth. How readily the armour of self-defence is donned, often before there is the slightest indication of attack. Carlyle says, most truly, the greatest of all faults is to be conscious of none.

Of course this hyper-sensitiveness is more common in some individuals than others, but we have reason to think that it has a special tendency to manifest itself in nursing communities, and if so—oh, the pity of it! The grand scope of the work marred by the details, the base materials wrought upon the beautiful foundation.

The cure is not difficult to find, but hard to enforce. What is wanted is sincerity that is not self-conceit. Those who are really sincere in the love of their chosen employment will forget themselves in the magnitude of the whole, and will be content to work on and live on unnoticed as integral parts of an infinite scheme.

### The Book World for Women and Nurses.

[We invite Correspondence, Criticism, Enquiries, and Notes on Books likely to interest Women and Nurses. Address, Editor, THE HOSPITAL (Nurses' Book World), 28 & 29, Southampton Street, Strand, London, W.C.]

A MANUAL OF HYGIENE FOR STUDENTS AND NURSES. Illustrated by seventy drawings. By JOHN GLAISTER, M.D., D.P.H. (Camb.) (London: The Scientific Press. 1897. Price 3s. 6d.)

As a general introduction to sanitary science this book is likely to prove very useful to not only those for whom it is more especially addressed, but to that large and increasing section of the general public who wish to know something of the general principles of hygiene. It is written in a flowing and easy style, and although it is somewhat discursive, touching on a vast variety of topics, it is perhaps for that very reason all the more interesting. A considerable portion of it originally appeared as a series of papers in the Nursing Section of THE HOSPITAL, when it attracted considerable attention. In its present form it makes a very useful manual. It is very well printed, and the illustrations are very good.

Y.W.C.A. SKETCHES. 1897. Edited by W. GRAHAM, Honorary Educational Secretary, and published under the direction of the British National Council. Price 1s.

This attractive little publication, being a record of the growth of the Young Women's Christian Association from 1855 to the Diamond Jubilee of her Majesty Queen Victoria, June 22nd, 1897, forms a very suitable Jubilee commemoration volume of the association. The growth and development of the work are carefully followed up in a series of nine short sketches dealing with every branch and bye-way of the association. The pages of the little book are brightened and made still more interesting by the noble and kindly faces of those connected with the movement from its early days to the present time. Of the latter two of the faces are especially noticeable from the great beauty of feature and expression, viz., that of Mrs. Pennefather, one of the first presidents, and that of Miss Clifford, of the Bristol Teachers' Branch. The publication not only contains useful information to those in any way connected with the association, but should be of interest to the general public, who may by reading it have their eyes opened to the great and far-reaching help so unobtrusively given to those weaker members of our great social body, who in default of it would go to the wall.

### Appointments.

### MATRONS.

HOSPITAL FOR EPILEPSY AND PARALYSIS, REGENT'S PARK.—On October 20th Miss Dorothea M. Oldham was elected Matron of the above hospital. She was trained at Addenbrooke's Hospital, Cambridge, and has previously been matron of Salop Infirmary, Shrewsbury, and for three months matron of the Epileptic Colony, Chalfont St. Peter. She has held appointments in the Midland Counties Home for Incurables, Leamington, and the General Hospital, Cheltenham.

THORPE FEVER HOSPITAL, EASINGTON, DURHAM.—Miss Alice Houldey was elected Nurse-Matron of the above hospital on October 21st, 1897. She was trained at the Sanitary Hospital, Taunton. The appointments she has successively held are as nurse at the same hospital, nurse at the North Cambridge Hospital, Wisbech, and head nurse Fever Hospital, Stockton.

SIR TITUS SALT'S HOSPITAL, SHIPLEY.—On October 6th Miss D. Williamson was elected Matron-Nurse of the above hospital. She received her training at the Northern Hospital, Liverpool, and has been sister of accident wards and operation theatre, the Northern Hospital, Liverpool.

### Minor Appointments.

CRAIGLOCKHART PAROCHIAL HOSPITAL, EDINBURGH.—Miss Jessie Dobbie, trained at Royal Longmore Hospital, Edinburgh, and at Western Infirmary, Glasgow, was appointed Staff Nurse at the Edinburgh Parochial Hospital, Craiglockhart.

GAINSBOROUGH WORKHOUSE.—Miss Jane Green was elected Head Nurse of this institution on October 26th. She was trained at Mill Road Infirmary, Liverpool.

### Where to Go.

AN EMBROIDERY EXHIBITION.—Lovers of needlework cannot fail to enjoy the exhibition of church embroidery at Messrs. Harris and Co.'s, 25, Old Bond Street. The firm have placed the selection of frontals, altar linen, stoles, &c., which were so much admired at Nottingham, on view by special request. The linens are almost equal to silk in appearance, whilst their durability is unrivalled. Dwellers in hot climates will be glad to know that linen fabrics are proof against the ravages of the ants. Messrs. Harris have also a variety of prepared work of great beauty, both in design and colour. Nurses are sure to appreciate the wonderfully effective appliqué work, as it lends itself readily to ward decoration. A banneret of the "Good Shepherd" will be sure to excite the admiration of those who have charge of children's wards and hospitals.

NORTH-EASTERN HOSPITAL FOR CHILDREN.—A bazaar and sale of work is being held at the parish room of St. John-at-Hackney, in aid of the North-Eastern Hospital for Children. The bazaar remains open until Friday, and can be visited from three to half-past ten each day. Various musical entertainments are provided.

### Presentations.

LAST month Sister Palmer was presented with a handsome marble clock from her night nurses at Gore Farm Hospital, Dartford, where she had occupied the position of Night Superintendent for a year, together with an address expressing their sorrow at her leaving them and their thanks for the good work she had done whilst among them. Miss Palmer, who was trained and certificated at Guy's Hospital, and also received the Butterworth Medal for five years' service there, has now been appointed as Housekeeping Sister at the New Park Fever Hospital, Hither Green, where she carries with her the best wishes of all her friends and fellow-workers for her success and happiness.

NUNEATON AND DISTRICT.—Nurse Watkins Pitchford (late district nurse in connection with the Nuneaton and District Cottage Hospital) was presented with a set of silver-backed brushes by five of the members of the medical staff of the district and hospital, upon her leaving after over two years' work.

NURSE TAITE, on resigning her post of District Nurse at Stonehouse, was presented with a gold watch, a silver sugar basin, and a travelling rug, by the doctor and patients for whom she worked.

### Notes and Queries.

**The contents of the Editor's Letter-box have now reached such unwieldy proportions that it has become necessary to establish a hard and fast rule regarding Answers to Correspondents. In future, all questions requiring replies will continue to be answered in this column without any fee. If an answer is required by letter, a fee of half-a-crown must be enclosed with the note containing the enquiry. We are always pleased to help our numerous correspondents to the fullest extent, and we can trust them to sympathise in the overwhelming amount of writing which makes the new rules a necessity. Every communication must be accompanied by the writer's name and address, otherwise it will receive no attention.**

*Probationer's Duties.*

(39) E. B. is reminded of our rule not to answer queries unless the name and address of the sender accompanies the question.

*Monthly Nursing.*

(40) (1) Will you please tell me through THE HOSPITAL how can a nurse get monthly cases after she has gained her certificate? (2) Also are there any associations employing nurses with only the monthly certificate?—*Elizabeth.*

As a beginner without interest the best plan is undoubtedly to join an association of good repute, and when you have made a name as a reliable and skilful nurse well known to several medical men, you may begin to form a private connection, and extend it by advertising.

*Superannuation Fund.*

(41) I am a certificated nurse, and have served fourteen years in workhouse hospitals. This past two years I have been private nursing for myself, during which time the above fund has been opened for Poor Law officials. If I entered a workhouse again as nurse, should I have to go on paying into the fund until I was 60, or could I pay into the fund (after entering the Poor Law service) for a quarter, and draw the pension when I was 60? (2) Would the pension depend on the highest salary I had ever received from the Guardians, or the salary I was in receipt of at the time I joined? I have heard after ten years' service it entitles me to a pension.—*Agnes Crawford.*

Sec. 4 of the Poor Law Superannuation Act of 1896 provides that "all service by an officer under any Poor Law authority shall be aggregated, whether continuous or not." Therefore, if you again take service under the Poor Law authorities, and pay into the pension fund until the prescribed age, your pension will be calculated on the total number of years you have served, including the fourteen years you have already completed. It is entirely to your advantage to return to this work.

*The Care of the Hair.*

(42) Could you let me know if hair wash I saw in your paper to Sister Gertrude is to be used when washing the hair with ordinary water or do you just rub it into roots, wait till it is dry, and then comb and brush hair as usual? (2) I should like to know what sort of oil is to be used if hair is dry? (3) Can you recommend anything for hair turning grey?—*Rita.*

Full directions were given for the use of the hair wash to Sister Gertrude. It is, perhaps, more correct to call it a head wash, and unless it be well dried off with a towel after application to the scalp the hair is not soft again for some days. If the hair wants washing nothing is more satisfactory than to plunge it into a bowl of new milk-warm soft water, and lathering freely with good soap, bathing the lather off in the same water. (2) Any fine oil is suitable, lanoline, maccassa, olive, neatsfoot. (3) No remedy has been discovered that prevents the hair turning grey. Care of the general health and absence of mental fret retard the process, but nothing checks it.

*Light Nursing.*

(43) I am 21 years of age, and have had some hospital experience, having undertaken holiday duty. I am not strong enough to enter a large hospital for training, but am very fond of nursing. Could you give me information as to a private nursing home or small fever hospital (seaside preferred), where I could receive a year's training? If required, I could pay a small premium.—*Nurse.*

A judiciously-worded advertisement would most probably help you most.

*Hospital Storekeeper.*

(44) Will you kindly inform me, through the columns of "Notes and Queries," if a situation as storekeeper in a hospital would be suitable for a young man having served 10 years in the grocery trade? In what papers are the vacancies advertised, and about what wages would he receive?—*E. M.*

The care of stores generally falls to the matron or steward, consequently it would be extremely difficult to hear of and obtain a post as storekeeper.

*Bicycle Saddle.*

(45) Will you kindly let me know the name of the bicycle saddle which you mentioned as strongly recommended by the medical profession in your HOSPITAL columns some weeks back? Is it suitable for a lady who has a very large district and many hours' riding?

(1) The Christy Anatomical Saddle. A. Spalding and Bros., 54, Holborn Viaduct, E.C. (2) So much depends upon the rider and the adjustment of the saddle that it is always well to try different saddles until you get one to suit.

*District Nurse.*

(46) Would you kindly inform me how I could best become a district nurse? I have answered several advertisements, but to no purpose. I possess a three-year certificate and good testimonials, and have done private work.—*A. Slack.*

Miss Peter, 1, St. Katherine's, Regent's Park, N.W., the superintendent of the Queen Victoria Jubilee Institute, advertised in the "Mirror" of October 9th for nurses for district work. You could not do better than apply.

## Figures and Tables

**Fig. 1. f1:**
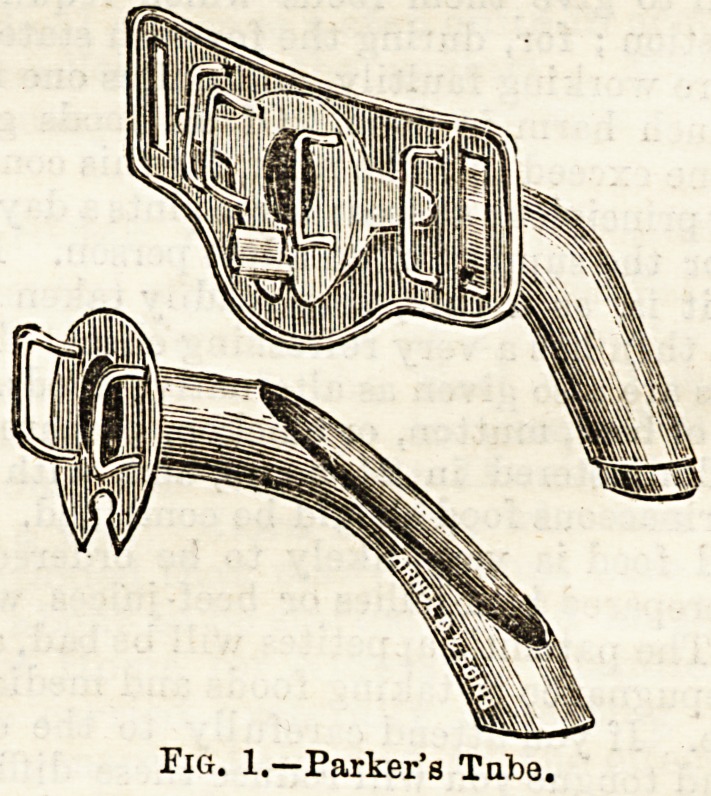


**Fig. 2. f2:**